# A Survey on NiTi Rotary Instruments Usage by Endodontists and General Dentist in Tehran

**Published:** 2011-11-15

**Authors:** Mohammad Ali Mozayeni, Amin Golshah, Nafiseh Nik Kerdar

**Affiliations:** 1. Department of Endodontics, Iranian Center for Endodontic Research, Shahid Beheshti University of Medical Sciences, Tehran, Iran.; 2. Department of Orthodontics, Dental School, Shahed University of Medical Sciences, Tehran, Iran.; 3. Department of Oral and Maxillofacial Radiology, Dental School, Jundishapur University of Medical Sciences, Ahvaz, Iran.

**Keywords:** Apical Transportation, File Fracture, Ledging, NiTi, Rotary Instruments, Usage

## Abstract

**INTRODUCTION:**

The aim of this study was to assess the extent of adoption, application and the associated issues with the nickel-titanium (NiTi) rotary instruments and techniques amongst endodontists and general dentists in Tehran.

**MATERIALS and METHODS:**

A total of 33 questions classified in six categories of demographics, frequency rate of NiTi rotary instrumentation and information. The sample size comprised of 100 endodontists and 100 general dental practitioners in Tehran.

**RESULTS:**

The overall response rate was 73.5%. NiTi rotary instruments were used by 98.4% and 50.6% of endodontists and general dentists, respectively. The main mentioned reason for not using rotary NiTi instruments was "lack of education". Among all procedural faults with NiTi, the most prevalent was “intra-canal file fracture” (88.5%) followed by "apical transportation" (71.2%) and "ledging" (68.3%). The main factors associated with the first procedural accident were "over-use" and “excessive pressure”.

**CONCLUSION:**

Dentists need more training and more comprehensive education regarding NiTi rotary instruments and techniques.

## INTRODUCTION

Cleaning and shaping of the root canal system is one of the main goals in endodontics which can be carried out using different systems and techniques [1]. To reach this aim, stainless steel hand instruments have been traditionally applied. Lack of flexibility of instruments causes errors during endodontic treatments [2] which lead to decreased success rate [3]. After introducing rotary nickel-titanium (NiTi), their usage became popular [4]. NiTi instruments super elasticity along with their advanced design made them favorable for effective and safe instrumentation of narrow and curved root canals using low torque handpieces [2]. The ability of some NiTi rotary systems in maintaining the root canal curvature has been studied [5][6][7][8][9][10]. Fracture susceptibility is considered as a major disadvantage of these instruments [1]. To date, there are a few studies about the adoption of this particular technology. A study which has been performed on using the Light speed rotary system in Switzerland showed that 80% of dentists used Lightspeed rotary system while 76% of those who used the system reported rotary instruments fracture experience [11]. Different reasons have been reported for instrument fracture such as excessive pressure, incorrect insertion angle and intra-canal complex anatomy. Recently, a questionnaire study in the USA showed that NiTi rotary instruments usage has correlation with region, graduation date and type of practice. More than 50% of respondents used NiTi rotary instruments for several patients before disposal; crown-down technique was found as the most frequent preparation method [12].

This study aimed to assess the extent of adoption, usage and issues associated with NiTi rotary instruments and techniques in endodontists and general dental practitioners in Tehran, 2009.

## MATERIALS AND METHODS

One hundred endodontists and 100 cluster sampled general dental practitioners of Tehran took part in this descriptive cross-sectional study. Using random cluster sampling with random tables, five clusters were selected as districts 3, 7, 11, 15, and 22. For selecting samples in each cluster, pen tip was blindly put on map on a particular spot considered as start point in cluster. Pen was moved in an anticlockwise Northward direction. In case if not enough samples were collected by finishing a block, the East, South and West blocks were also surveyed. 40 samples were selected in each district

A questionnaire was used for collecting information to describe, compare, or explain demographic characteristics, knowledge, attitude, preference, opinion as well as practical and experiences of the respondents. A total of 33 questions in both closed and open formats were included in the questionnaire. The structure of the questionnaire is described below:

Part A- Demographics (2 questions); Part B- Frequency rate of NiTi rotary instruments use (4 questions); Part C- NiTi rotary instruments using patterns (11 questions); Part D- Issues associated with NiTi rotary instruments use (10 questions); Part E- Issues associated with education about NiTi rotary instruments (4 questions); and Part F- Information about NiTi rotary usage (2 questions).

The questionnaire was designed and then evaluated by an endodontist and a medical research expert. To standardize the questionnaire, a pilot study was performed on 10 volunteer post-graduate students of endodontics. Their answers were obtained two times in 72-96 h. To evaluate two choice questions, Kappa test was used calculated at 0.75-1. For other questions, corresponding two answers was used with min of 81% and max of 100%. There was neither time limitation nor true/false question.

Endodontists and general dental practitioners who were working in Tehran were enrolled in study. Questionnaires with more than one third of non-answered questions were excluded. Respondents signed informed consent before enrollment and the study protocol was approved in ethics committee of Shahid Beheshti University of Medical Sciences. Answered questioners were collected during 6 months. The study was performed within November 2008 to December 2009.

Data were analyzed using Chi-square test, Fisher's exact test and Man-Whitney U test in Statistical Package for the Social Sciences Version14 (SPSS Inc., Chicago, IL, USA). Logistic regression analysis was used to confirm significant effect of several variables on NiTi rotary usage differences between endodontists and general dental practitioners. Significance level was set at P<0.05.

## RESULTS

### Part A. General and demographical data

This study achieved to an overall response rate of 73.5%. From 147 respondents of the current survey, 95 (64.6%) were male and 52 (35.4%) were female. Eighty five were (57.8%) general dentists while 62 remained (42.2%) were endodontists. Average age was 37.8±6.3 years for general dentists and 40.0±9.3 years for endodontists (ƒ=10.297 P=0.002), while work experience was 10.5±5.5 years and 12.3±7.8 years for two aforementioned groups respectively. The differences between two groups were not statistically significant.

### Part B. Frequency rate of NiTi rotary usage

Total of 104 (70.7%) respondents (43 general dentists and 61 endodontists) mentioned that they used rotary NiTi instruments (Table 1). Nonetheless, 43 (29.3%) have not used NiTi instruments due to lack of adequate education (46.7%), availability of hand instruments (46.7%), no perceived advantage (37.8%), non-availability of NiTi instruments (13.3%). Most of respondents who used NiTi instruments had more than one year of experience (Table 2). This characteristic was significantly higher in endodontists than general dental practitioners (Man-Whitney U test, z=3.354, P=0.001).

**Table 1:** NiTi rotary instruments usage according to the type of practice

****	**GD a(%)**	**Eb(%)**	**Total (%)**
**Use**	43(50.6)	61(98.4)	104 (70.7)
**Do not use**	42(49.4)	1(1.6)	43 (29.3)

^a^ General Dentists

^b^ Endodontists

**Table 2:** Experience with NiTi rotary instruments

**Period**	**GD^a^ (%)**	**Eb (%)**	**Total (%)**
**< 1 yr**	17 (39.5)	7 (11.5)	24 (23.1)
**2-3 yrs**	16 (37.2)	25 (41)	41 (39.4)
**< 3 yrs**	10 (23.3)	29 (47.5)	39 (37.5)

^a^ General Dentists

^b^ Endodontists

Among all dentists, 38.5% have treated 6-10 teeth/week; NiTi instruments were mostly used for molar teeth (91.3%) followed by anteriors (49%) and premolars (45%). The difference was not statistically significant between two groups for anteriors and molars. However, endodontists used NiTi instrument significantly more than general dental practitioners in premolars (X^2^=7.234, P=0.007). Straight canals (77.9%) were more commonly treated with NiTi instruments compared with curved canals (85.6%). Nevertheless, this characteristic did not reveal significant difference (straight canals: X^2^=0.511 P=0.909; curved canals: X^2^=0.013, P=0.909). NiTi instrument usage for coronal one third of root canals were more common in endodontists compared with general dentists (X^2^=14.313 P<0.001). However, this difference was not significant for one third of apical part (X^2^=2.166, P=0.141).

### Part C. Patterns of NiTi rotary instruments usage

*Technique:* General dentists mostly used Profile (46.5%), Mtwo (30.2%), and Flex Master (27.9%) systems. However, Flex Master (37.8%), ProTaper (32.8%), and Hero (29.5%) systems were accordingly the most common reported instruments by endodontists. Profile instruments usage was significantly different between two groups (P=0.012). Most of respondents (69.8% of general dentists and 72.1% of endodontists) used rotary instrument only with angles. Crown-down technique (85.6%) was the most common method following by Step-back method (39.4%). Most of respondents (69.8% of general dentists and 49.2% of endodontists) used Gates Glidden burs in the coronal one third of root canals and NiTi instruments in the apical one third (X^2^=16.151, P<0.001). From the population of this survey, 31.7% (18.6% of general dentists and 41% of endodontists) usually used hand instruments to prepare the apical part and NiTi instruments to prepare the coronal part (X^2^=5.831, P=0.016). However, 36.9% of respondents (25.6% of general dentistsand 27.9% of endodontists) uses NiTi instruments in both coronal and apical one third parts (X^2^=0.067, P=0.796). Furthermore, 26.9% used hand instruments for both apical and coronal parts. of three mentioned motors applied for driving instruments, DENTSPLY was notably used by general dentists (20.9%) and Endo It was widely used by endodontists (26.2%).

*Instruments reuse:* Among all responders, 25.6% of general dentists and 36.1% of endodontists answered 6-10 times. Also, 30.2% of general dentists and 23% of endodontists indicated 2-5 times and none mentioned single-use of these instruments. Twenty six percent of respondents used the files according to manufacturer’s instruction while 16.3% used these instruments until distortion. NiTi rotary instruments disposal decision was identified as after number of instrument reuse which mentioned in previous question (56.7%), after file distortion (41.3%), after decreased cutting efficiency and inability to be cleaned (18.3%), after use in curved canals (11.5%), after file fracture (10.6%), and after use in narrow canals (7.7%).

*Retreatment:* that the results of this study indicated that 96.1% of our dentists retreated root canals. Most of dentists (54.8%) sometimes used NiTi instruments to remove gutta-percha, while 28.8% always did so. A minority of dentists (12.5%) never used NiTi instruments to remove gutta-percha.

### Part D. Issues associated with NiTi usage

*Procedural experience:* Procedural problems in NiTi rotary instruments and hand instruments by the respondents of our study are demonstrated in Table 3. Among the evaluated data, binding of the file in root canals by hand instruments was the only procedural problem which showed significant difference between endodontists and general dentists (X^2^=6.975, P=0.008) (Table 4).

**Table 3:** Procedural problems with NiTi rotary instruments and hand instruments

**Procedural Problems**	**General Dentists (%)**		**Endodontists (%)**		**Total (%)**	
	**NiTi**	**Hand**	**NiTi**	**Hand**	**NiTi**	**Hand**
**Ledging of the canal**	29 (67.4)	37 (86)	42 (68.9)	55 (90.2)	71 (68.3)	92 (88.5)
**Transportation of the canal terminus**	31 (72.1)	34 (79.1)	43 (70.5)	54 (88.5)	74 (71.2)	88 (84.6)
**Strip perforation of a curved canal**	24 (55.8)	35 (81.4)	27 (44.3)	50 (82)	51 (49)	85 (81.7)
**Straightening of curved canals**	24 (55.8)	34 (79.1)	33 (54.1)	51 (83.6)	57 (54.8)	85 (81.7)
**Excessive dentine removal**	28 (65.1)	28 (65.1)	42 (68.9)	34 (55.7)	70 (67.3)	62 (59.6)
**Binding of the file in the canal**	27 (62.8)	25 (58.1)	40 (65.6)	29 (47.5)	67 (64.4)	54 (51.9)
**File fracture**	38 (88.4)	33 (76.7)	54 (88.5)	42 (68.9)	92 (88.5)	75 (72.1)
**File overing**	35 (81.4)	32 (74.4)	43 (70.5)	40 (65.6)	88 (84.6)	72 (69.2)

**Table 4:** Comparing procedural problem with NiTi rotary instruments and hand instruments

**Problem**	**NiTi**		**Hand**	
	**X^2^**	**p**	**X^2^**	**p**
**Ledging of the canal**	0.023	0.879	0.419	0.547
**Transportation of the canal terminus**	0.322	0.570	0.649	0.421
**Strip perforation of a curved canal**	1.347	0.246	0.006	0.941**a**
**Straightening of curved canals**	0.030	0.863	0.348	0.555
**Excessive dentine removal**	0.160	0.689	0.921	0.337
**Binding of the file in the canal** ****	0.085	0.770	6.975	0.008
**File fracture**	0.001	1.000**a**	0.781	0.377
**File overing** ****	1.599	0.206	0.926	0.336

^a^ Fisher's exact test

*Instrument fracture:* NiTi file fracture experience was reported by 83.7% of general dentists and 88.5% of endodontists. This characteristic did not reveal statistically significant difference between two studied groups (X^2^=0.500, P=0.480) (Table 5). General dentists experienced more file fracture at sizes 20 and 25 with 0.02 and 0.04 taper; whereas endodontists reported most file fractures at sizes 25 and 30 with 0.04 and 0.06 taper. Profile (25.6%), K3 (23.3%), Flex Master (16.3%), ProTaper and Mtwo (14% for each) in general dentists and Flex Master (34.4%), Profile (29.5%), Hero (21.3%), Mtwo (19.7%), and Race (18%) in endodontists were the most common fractured files. Flex Master and Hero file fracture was significantly higher in endodontists than general dentists (P<0.05). File fractures mostly occurred at the third apical part of root canals. This characteristic did not achieve a significant difference between two groups (X^2^=1.357, P=0.507). File fracture was most common in the apical part (76.9%) following by middle part of canal (26.9%). This procedural accident was rarely reported in the coronal third part of root canals (3.8%). No significant difference was achieved between two groups for this characteristic (X^2^=0.468, P=0.791). A detailed breakdown of identified factors associated with file fracture is presented in Table 6.

In case of fracture, most of respondents reported retrieving the fractured file (62.5%). A considerable number of respondents (51.9%) obturated root canal only with reviewing the position of fractured file in the canal. Only a few referred such patient to an endodontist (19.2%). Referring to an endodontist was the only characteristic statistically significant differences between two groups (X^2^=11.564, P=0.001). Neither in general dentists nor in endodontists was extraction reported as an option for teeth with fractured files.

**Table 5:** Incidence of file fracture for GD and E^a^

**Number of files fractured**	**GD^b^ (%)**	**E c(%)**	**Total (%)**
**1-5**	15 (34.9)	19 (31.1)	34 (32.7)
**6-10**	11 (25.6)	13 (21.3)	24 (23.1)
**11-15**	7 (16.3)	9 (14.8)	16 (15.4)
**>15**	6 (14)	13 (21.3)	19 (18.3)
**No response**	4 (9.3)	7 (11.5)	11 (10.6)

^a^ Man-Whitney test z=0.726, p=0.468

^b^ General Dentists

^c^ Endodontists

**Table 6:** Reported reasons for file fracture by GD and E

**Reason**	**GD^a^**		**Eb**		**Total**	
	**Frequency**	**%**	**Frequency**	**%**	**Frequency**	**%**
**Excessive pressure on file**	24	55.8	46	75.4	70	67.3
**Incorrect insertion angle of file**	7	16.3	15	24.6	22	21.2
**Nonconstant speed of rotation of file**	4	9.3	8	13.1	12	11.5
**r.p.m. too high**	3	7.0	5	8.2	8	7.7
**No irrigant in canal**	10	23.3	5	8.2	15	14.4
**Incorrect file sequence**	11	25.6	6	9.8	17	16.3
**Complex root canal anatomy**	21	48.8	22	36.1	43	41.3
**Over-usage**	28	65.2	48	78.7	76	73.1
**No usage of motor with appropriate torque **	10	23.3	6	9.8	16	15.4
**Type of file**	6	14.0	6	9.8	12	11.5
**Unknown**	2	4.7	4	6.6	6	5.8

^a^ General Dentists

^b^ Endodontists

### Part E. NiTi education

Overall, 55.8% of general dentists and 42.4% of endodontists attended NiTi rotary instruments complementary training courses which was not statistically different (X^2^=1.8, P=0.18). Among respondents who attended these postgraduate programs, 45.8% of general dentists and 44% of endodontists mentioned that they used NiTi rotary instruments before the courses (X^2^=0.017, P=0.897). Effectiveness of training courses was reported low, intermediate, and high by 37.5%,33.3%, and 29.2% of dentists. Evaluating results for endodontists, these were very low 8%, low 16%, medium 64%, and high 12%. In general, 89.5% of dentists and 96.6% of endodontists recommended NiTi usage. A detailed breakdown of mentioned advantages of these instruments is shown in Table 7.

**Table 7:** Reported benefits by general dentists (GD) and endodontists (E)

**Benefit**	**GD (%)**	**E (%)**	**Total (%)**
**Maintain canal curvature**	19 (44.2)	43 (70.5)	62 (59.6)
**Faster canal preparation**	26 (60.5)	39 (63.9)	65 (62.5)
**Maintain working length**	8 (18.6)	16 (26.2)	24 (23.1)
**Easier final canal obturation**	13 (30.2)	21 (34.4)	34 (32.7)
**Simplicity for dentists and patients**	26 (60.5)	46 (75.4)	72 (69.2)
**Easier obturation of teeth hardly available**	17 (39.5)	28 (45.9)	45 (43.3)
**Easier and faster canal retreatment**	16 (37.2)	27 (44.3)	43 (41.3)

Finally, a logistic regression analysis was performed with all variables that were statistically significant in order to establish the most important variables on NiTi instruments rotary usage differences between endodontists and general dental practitioners (Table 8). Referring to an endodontist was higher for general dental practitioners. Hero file fracture was higher for endodontists. General dental practitioners more used Profile instruments files. Binding of the file in root canals by hand instruments more accrue for general dental practitioners. Endodontists experienced more Flex Master file fracture.

**Table 8:** Result of logistic regression for significant NiTi rotary instrument usage differences between groups

**Significant variables**	**OR **	** SE**	**Wald **	** Sig.**
**Profile instruments usage**	2.68	0.14	67.34	P<0.05
**Hero file fracture**	3.09	0.12	46.65	P<0.05
**Flex Master file fracture**	1.34	0.10	78.23	P<0.05
**binding of the file in root canals by hand instruments**	1.67	0.10	123.35	P<0.05
**Referring to an endodontics**	7.87	0.19	35.42	P<0.05
**NiTi instrument usage for coronal one third of root canals**	0.21	0.15	54.13	NS******

## DISCUSSION

The results of this study showed that 70.7% of respondent (98.4% of endodontists and 50.6% of general dentists) used NiTi rotary instruments. Our findings were in consistent with some previous studies: 22% of general dentists and 64% of endodontists in an Australian study [1], approximately 70% of general dentists and almost 83% of endodontists in a study performed in UK [13][14] as well as 77% of the Swedish general dentists who participated in an endodontics educational program [15] have mentioned that they used NiTi rotary instruments.

It was previously identified that the average teeth treated per week is in direct relation with a NiTi rotary instruments adoption [16][17]. Accordingly, 38.5% of the respondents of our study used NiTi instruments for treatment of 6- 10 teeth/week; which was in line with Parashos and Messer, and Madarati et al. [1][13][14] but was in contrast with the results of a previous study [11]. This difference might be attributed to the different type of rotary instruments usage, since Light-Speed technique, was not used in our study.

In accordance with previous reports [1][12], crown-down was the most common technique for canal preparation. However, it should be noted that dentists have employed sequence of NiTi rotary and hand instruments according to clinical conditions. Majority of dentists used NiTi instruments for 6-10 times; mostly based on serviceability of the instrument. Parashos and Messer have demonstrated that 70% of dentists used NiTi for 2-5 times; among which, 84% noted serviceability as the main criterion for application [1]. In contrast, a study by Madarati et al. [13][14] in UK showed that 44.8% of respondents discarded instruments after a single usage. This characteristic might indicate the responsibility in number of uses in UK practitioners. File fracture (88.5%), file covering (84.6%), Transportation (71.2%) and Ledging formation (68.4%) have been demonstrated as the most common procedural accidents with NiTi rotary instrument. Ledging formation(88.5%), transportation of the canal terminus(84.6%), strip perforation (81.7%) and straightening curved canals (81.7%) were the most frequent procedural accidents in hand instruments reported in this assessment. These results are in line with some of previous articles [18][19]. However, there is one report which demonstrated that there was no significant difference in file fracture between users of NiTi rotary instruments and hand instruments [19]. Additionally, similar to Madarati et al. [13][14], our study showed that instruments’ fracture was higher in endodontists than general dentists.

Profile and K3 in general dentists and Flex Master and Profile in endodontists were the most common fractured files. In previous reports [19][20] ProTaper was reported as the most common fractured file. In our study, the incidence of file fracture for Profile, ProTaper, GT Rotary and K3 Endo showed no statistically significant difference.

General dentists experienced fracture of files at sizes 20 and 25; whereas, for endodontists file fracture happened most at sizes 25 and 30. In a previous report, Guelzow et al. [19] showed the most file fracture at size 30. Di Fiore et al. [20] reported the tip sizes of the instruments that fractured ranged from 20 to 40.

Factors assumed to be responsible for file fracture (Table 6) revealed that dentists mostly inclined to find the mechanisms underlying file fracture. Over usage (73.1%), excessive pressure on file (67.3%) and complex root canal anatomy (41.3%) were the most common assumed responsible factors for file fracture by the respondents of our survey. This list has been designed after Barbakow and Lutz study [11] revealed that excessive pressure on file (25%), incorrect insertion angle (17%), and complex root canal anatomy (15%) were the most common reported associated factors. Also, Parashos and Messer [1] identified excessive pressure on file (62%), over usage (43%), and complex root canal anatomy (36%) as the most frequent reasons for file fracture. In the Madarati et al. study [13][14]; main factors were related to the operator (i.e. experience, frequency of instruments usage. This discrepancy is likely due to using different instruments and techniques. In cases of file fracture, our results are almost similar to earlier studies [1][13][14].

In our study, dentists and patients comfort (69.2%), faster canal preparation (62.5%), and maintaining canal curvature (59.6%) were the main advantages of using NiTi rotary instruments. In comparison to a study by Parashos and Messer [1], faster canal preparation (80%), maintaining canal curvature (73%) and easier final canal obturation (72%) were identified as the most important advantages. However, in a study by Barbakow and Lutz [11], safety (82%), dentists and patients’ comfort (76%) and faster canal preparation (54%) and in a study by Bjourndal and Reit [21] faster canal preparation, consequently decreased visit sessions and treatment length were the most reported advantages. Koch et al. [15] reported greater root filling quality, less physically tiring technique for practitioners along with fast and easy procedures as advantages. Because of shorter treatment length, most patients are likely to refer to endodontists in comparison to general dentists [16]. Our study findings and other published literature [1][11][19][22], support the idea of shortening treatment length using NiTi rotary instruments. It can be suggested that general dentists could apply new techniques to shorten treatment length.

In the current survey, using NiTi instruments increased with increasing work experience (23.1% <1 yr, 39.4% 2-3 yrs, and 37.5% >3 yrs), which is in agreement with previous observations [1][12]. This would suggest that dentists with lower work experience, for some reasons, are less likely to use NiTi instruments. One reason could be described as younger dentists are likely to improve their experience as regard to hand instruments, before applying new techniques which require specific education and training. Another reason can be explained that these groups of dentists think these techniques take them too much time to be learned. Hence, they prefer to stick with their traditional technique.

One of the main obstacles to use NiTi instruments by dentists lies in unbelieving in new techniques [1][17]. Along with the latter, beliefs that NiTi rotary instruments are prone to fracture and dealing with them is complicated, are of causes that dentists do not use these instruments routinely. In addition, a large scale of dentists believe that it takes them too much time to learn how to work with NiTi rotary instruments, which might reveal that they are widely under the influence of their senior colleagues [23][24][1][17]. In our study, reasons for not using NiTi instruments appear the same as the ones which have been previously reported [1][15]. The most important reason for not using NiTi instruments seems to be lack of adequate education.

In our study, 24 dentists and 25 endodontists attended complementary training courses of which 11 general dentists and 11 endodontists had used NiTi instruments before training. In Reit et al. study [17] only 4% of dentists used NiTi instruments before attending courses. However, after attending theoretical and practical courses this quantity increased to 53% and 94% respectively. These courses might have positive/negative points. Positive points include the necessity for acquaintance of dentists with new techniques. Negative points considered by some dentists is that runners of these courses mainly aim at selling their products. This suggests that considerable attention should be given to distribution of new techniques and instruments [1]. In Parashos and Messer study [1], only 30% of dentists attended "university" training courses implying that universities are not adequately engaged in familiarizing dentists with new techniques and more pessimistically are not familiar with the needs of their dentists, since their reliance is on endodontists who themselves may not be fully aware of new technology. In our study, 86.5% of those who had experienced file fracture suggested NiTi instruments to their colleagues.

Finally, according to present study and some others [25][1] it should be highlighted that training courses are necessary for using NiTi instruments. These courses should be more comprehensive and without bias by professionals familiar with a specific new technology.

## CONCLUSION

Dentists are familiar with limitations of NiTi instruments and techniques. Moreover, they are increasing efficacy of their practice by using these appliances. Current study showed the awareness of dentists about benefits of NiTi rotary instruments application comparing to traditional techniques and also the high percent usage of these instruments among endodontists and general dentists. Results of this questionnaire have demonstrated that dentists and dental students need more training and more comprehensive education regarding new techniques and methods.
